# Rapid adoption of bow technology across western North America ∼1,400 years ago

**DOI:** 10.1093/pnasnexus/pgag040

**Published:** 2026-03-17

**Authors:** Briggs Buchanan, Marcus J Hamilton, Metin I Eren, Robert S Walker

**Affiliations:** Department of Anthropology and Sociology, University of Tulsa, Tulsa, OK 74104, USA; Department of Anthropology, University of Texas at San Antonio, San Antonio, TX 78249, USA; Santa Fe Institute, Santa Fe, NM 87501, USA; Department of Anthropology, Kent State University, Kent, OH 44242, USA; Cleveland Museum of Natural History, Cleveland, OH 44106, USA; McDonald Institute for Archaeological Research, University of Cambridge, Cambridge CB2 3ER, United Kingdom; Department of Anthropology, University of Missouri, Columbia, MO 65211, USA

**Keywords:** weapon technology, organic preservation, radiocarbon dates, optimal linear estimation, Bayesian logistic regression

## Abstract

The replacement of the atlatl and dart by the bow and arrow marks a major technological transformation in the human past, yet the timing and dynamics of this transition in North America remain poorly resolved due to the poor preservation of organic weaponry. Here, we compile and analyze a dataset of 140 radiocarbon dates from 136 well-preserved organic weapons recovered from western North America, spanning approximately the last 10,000 years. Using chronological modeling, optimal linear estimation, and Bayesian logistic regression, we show that bow technology first appears in both northern and southern regions around 1,400 years before present. The dynamics of adoption, however, differ sharply by region. In the south, across a vast area from northern Mexico to California and the Southwest, the bow rapidly and almost completely replaces the atlatl, a case of technological disruption in which an innovation decisively renders an older system obsolete. In the north, in contrast, the bow and atlatl coexisted for more than a millennium. This coexistence reflects a broader global pattern of increasing technological richness at higher latitudes, where ecological risk is mitigated through diversification rather than specialization. Our findings clarify previous claims of much earlier bow use in North America, demonstrate the importance of rare invention events followed by rapid diffusion, and highlight the contingent pathways by which technologies are adopted or abandoned. These results provide insight into the processes of technological evolution, showing how innovation, ecology, and cultural transmission interact to shape long-term human history.

Significance Statement A transition from the atlatl to the bow is one of the most important technological shifts in human prehistory, reshaping hunting and conflict. Using directly dated organic weapons, we show that bow technology emerged nearly instantaneously across western North America 1,400 years ago, but the adoption dynamics differed between southern and northern regions. In the south, the bow displaced the atlatl in a disruptive transition, while in the north, the two technologies coexisted for over a millennium. Our findings highlight how human societies navigate adaptive problems through different strategies depending on the innovation and the ecological and social contexts into which they are introduced, sometimes specializing by adopting a single technology and at other times diversifying by maintaining multiple solutions.

## Introduction

Humans and their hominin ancestors have been using technology to solve problems of energy capture presented by the environment for at least 3.0 million years (1, 2). Archaeological and historical records document that as technologies evolve, older technologies can become obsolete and be replaced by newer innovations (3, 4). The archaeological record provides the deep time perspective to study the process of technological evolution in the human past.

Here, our focus is on weaponry technology used by small-scale societies. A striking case of technological innovation is when bow technology replaces atlatl technology. The bow and arrow represent a weapon system that harnesses a novel configuration of mechanical properties, drawing on the power of elasticity to transfer kinetic energy to an arrow, as opposed to the lever action of the atlatl (5). Generally, relative to atlatl technology, bow technology confers several advantages in projectile delivery, including increased arrow accuracy, distance, and velocity; increased shots per unit time; reduced spatial requirements to make a shot; increased stealth; and the ability to reload and shoot from a variety of positions (e.g. from horseback or a tree, or kneeling, crouching, or lying down) (6–14). Yet, there are costs to bow technology too, such as increased production and maintenance costs and complexity, decreased ability to deliver large weapon tips, use of both hands for bow operation precluding or hindering use of a defensive shield, and inhibited bow use in certain weather conditions (11, 14). Thus, in cases of replacement, the benefits of the new technology must have outweighed the costs of adoption within a particular ecological and social context (15).

The adoption of bow technology has long been of interest to archaeologists around the world (16–30) given its introduction occurred independently in several different prehistoric geo-temporal contexts, such as South Africa (17, 24), Central Europe (27, 30), Western Europe (26), South Asia (23), and North America (16, 18, 29). As such, bow technology commonly serves as a case study of prehistoric technological innovation, diffusion, and obsolescence (31–36). However, determining the timing of the introduction and spread of bow technology is made difficult by the fact that atlatls, darts, bows, and arrows are composed primarily of perishable organic materials, apart from tips and atlatl hooks made of stone, bone, or metal. As such, these technologies do not preserve well in the archaeological record unless recovered in rare environmental settings that promote preservation, such as dry caves and rock shelters, anaerobic sites, and ice sheets. Commonly, only the stone or metal weapon tips are preserved in the archaeological record, and so archaeologists use indirect methods to infer the type of weapon delivery system used in the past. These methods include the examination of impact features and residues on tips but often focus on using the form and weight of weapon tips to distinguish between spear, dart, and arrow technologies (9, 22, 29, 37, 38). While using stone point form has the advantage of drawing from large samples of preserved weapon tips in the archaeological record, there is always uncertainty in identifying the type of weapon delivery system with which points were used.

In this paper, we examine well-preserved organic weapons where the weapon delivery system—atlatl or bow—can be identified with minimal uncertainty. When radiocarbon-dated, well-preserved organic weapons provide direct evidence of when and where a weapon system was in use. Across western North America, ideal preservation conditions include ice sheets, dry caves, and rock shelters from Canada and Alaska to northern Mexico. A particularly abundant region for preserved weapon systems is the Yukon and Northwest Territories, where archaeologists have recovered a large sample of prehistoric organic remains along the recently melted peripheries of glacial ice (39–42). Grund and Huzurbazar (21) used a sample of directly dated weapons from this region to investigate the timing of the transition from atlatl to bow. They found that the bow first appeared around 1,200 cal.BP and overlapped in time with atlatl technology for 174 ± 135 years. Our study expands on this work by generating a new database of dated organic weapons across western North America to examine the adoption dynamics of the transition between atlatl and bow technologies.

The timing of this replacement in western North America has been the subject of much research. Documented use of the atlatl and dart in the far north and in Mesoamerica at the time of European contact brackets this large region of the continent with cases of potentially long temporal overlap of the atlatl and bow (43). While atlatl technology most often predates bow technology (39–42), estimates of the first appearance of bow technology in the west are wide ranging. Blitz (18) proposed a temporal gradient for the diffusion of bow technology across North America originating in the far north by ∼5,000 cal.BP and diffusing south across the Great Basin between 1,800 and 1,500 cal.BP and arriving in the Southwest by 1,400 cal.BP. Maschner and Mason (25) argue that bow technology was present in the far north by 12,000 cal.BP, while Amick (44) has suggested that Folsom peoples of the Southwest, Rockies, and Plains regions were using bow technology during the late Pleistocene. Ames et al. (16) argue that bow technology was in use in the Northwest by 8,500 cal.BP, and other researchers working in the Great Basin and Southwest have suggested several other alternative dates (32, 34, 36, 38, 43, 45–47). As such, the nature of the timing and adoption of bow technology remains an important question that has not been adequately addressed using a comprehensive database of dated, well-preserved organic weapons.

Here, we use a dataset of preserved, dated weapon systems to examine these invention and adoption dynamics. In the north, our sample of weapons comes from receding glacial ice patches, and in the south, our sample comes primarily from dry caves and rock shelters in southwestern North America. Together, these provide an ∼10,000-year window into the use of organic weapon technologies. We use chronological modeling to examine the timing of the transition and optimal linear estimation (OLE) to estimate the origin of the bow and arrow. We show that bow technology appears around 1,400 years ago in both regions, but the process of transition is different in the two regions; in the north, atlatl technology continues to be used for more than 1,000 years after the introduction of bow technology, whereas in the south, the transition to the bow occurs rapidly and completely replaces the atlatl nearly instantaneously. Our findings revise current understandings of the appearance of bow technology and the process of technological transition in both regions; the temporal overlap between the two technologies in the north was previously underestimated, whereas in the vast area covered by the southern region, there is no evidence of temporal overlap, suggesting a process of technological disruption. The continued use of the atlatl technology with bow technology in the north fits the global pattern of increasing richness in technological complexity in northern latitudes.

## Results

**Fig. 1.**
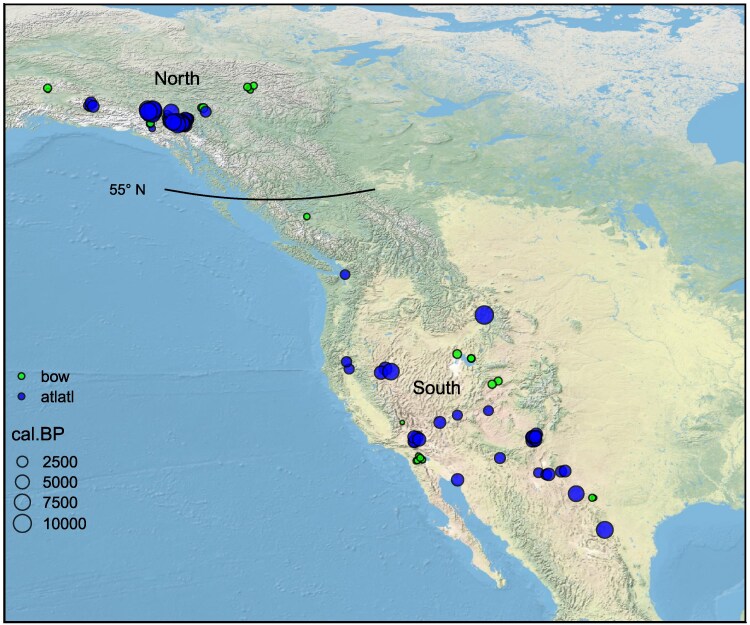
Locations for the 86 atlatl and dart and 50 bow and arrow radiocarbon-dated weapon systems used in this study. The size of each circle corresponds to the calibrated calendar date in years before present (cal.BP). The sample is split at 55° north latitude, with specimens >55° considered the north sample and specimens <55° considered the south sample.

**Fig. 2.**
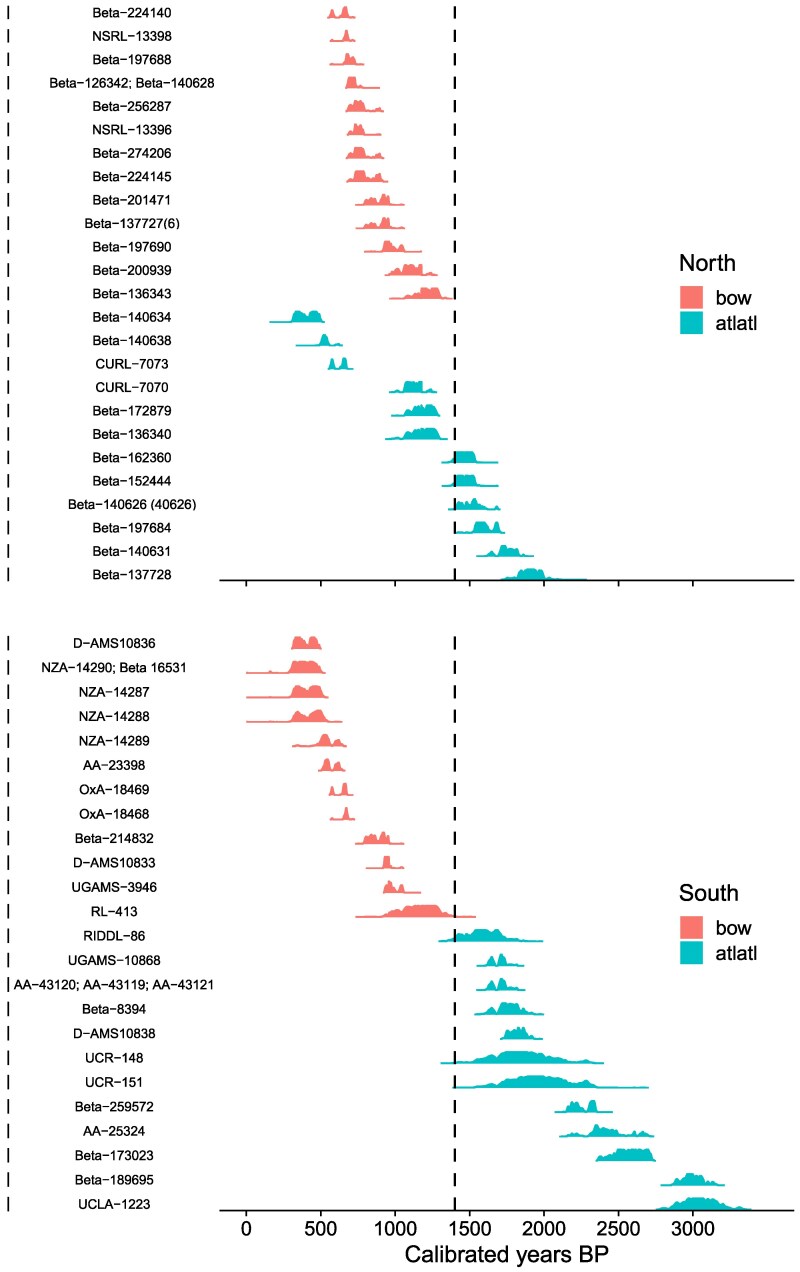
Twenty-four calibrated radiocarbon dates associated with atlatls and darts (turquoise) and bows and arrows (red) transition in the (top) north and (bottom) south regions. This subsample of individual radiocarbon assays is identified along the *y*-axis by their lab and sample number. Multiple lab and sample numbers for individual assays indicate multiple radiocarbon dates from the same specimen that have been averaged. Sample numbers in parentheses indicate published discrepancies.

The geographic distribution and approximate ages of the 136 atlatl and bow samples from the north and south regions are plotted in Fig. 1. In Fig. 2, we plot the 24 dates (12 dates from atlatls or darts and 12 dates from bows or arrows) closest in age to the overlap between these technologies for the north and south regions. Figure 2 shows that bow technology appears around 1,400 years ago in both regions. It is also evident that the time it takes for the transition differs between the north and the south. In the north, the atlatl continues to be used for nearly 1,000 years after bow technology first appears, whereas in the south, the first appearance of bow technology marks the almost immediate replacement of the atlatl.

Summarizing the radiocarbon dates for atlatl and dart and bow and arrow by region using the end-to-end Bayesian analysis shows the overlap between atlatl and bow technology in the north and the rapid replacement of the atlatl in the south (see Supporting Information and Fig. S2). The OLE estimates for the origin of the bow in the south range from 1,019 cal.BP to 1,818 cal.BP with a mean of 1,396 cal.BP. For the north, the OLE estimate for the origin of the bow has a mean of 1,430 cal.BP with a narrower range of 1,176 cal.BP to 1,651 cal.BP. The average OLE estimate for the origin of the bow for both regions is about 1,400 cal.BP and closely aligns with the timing of the transition identified in the summarized radiocarbon dates.

**Fig. 3.**
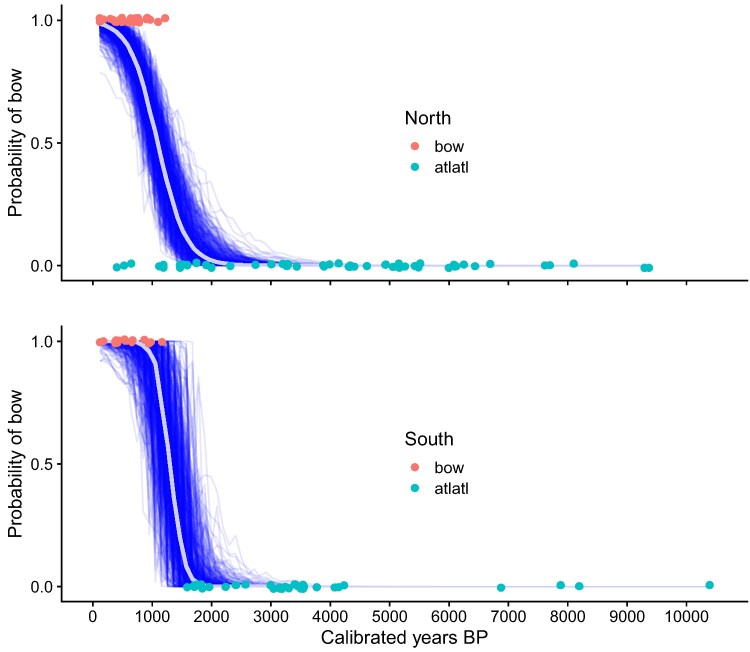
Bayesian logistic regression modeling of the probability of bow and arrow versus atlatl and dart use through time. Individual median ages of calibrated radiocarbon assays of bow or arrows (in red) and atlatls or darts (in turquoise) are shown. Five hundred logistic regression draws from the posterior are shown with blue lines and the overall average logistic curve is in gray.

Lastly, we used Bayesian logistic regression to model the timing of the transition from atlatl to bow in the north and south (Fig. 3). The slope of the logistic curve in the south is much steeper than it is in the north, around 1,400 years cal.BP when atlatl technology use switches to bow technology.

## Discussion

Our analysis of the transition from atlatl to bow technology in western North America shows that bow technology emerges in both regions ∼1,400 years ago. However, the rate of the transition between the two regions differs. In the north, the coexistence of the atlatl and bow for several centuries suggests that the atlatl continued to be used after the introduction of the bow. In contrast, in the south, across a vast area of western North America, the rapid disappearance of the atlatl following the introduction of the bow indicates a disruptive technological replacement in which a novel technological innovation quickly rendered the existing technology obsolete.

Our findings refine long-standing questions regarding the innovation and adoption of bow technology in North America. First, our results indicate that bow technology appears ∼1,400 years ago in both the north and south. This timing suggests that if bow technology was used in the North American late Pleistocene or early Holocene, as has been postulated (16, 25, 44), then it was likely a prior, independent occurrence. Our results are also inconsistent with the timing of Blitz's temporal gradient model (33). Instead, the evidence we present here indicates a relatively late introduction that occurred nearly simultaneously across a vast area, followed by regionally distinct adoption trajectories. If there was a single invention of bow technology in time and space, our study shows that the current resolution of the archaeological record is unable to identify such a specific, singular event. Indeed, we argue that the empirical observation that bow technology appears almost simultaneously in both regions around 1,400 cal.BP is likely explained by a single origin followed by rapid diffusion. While independent invention is theoretically possible, and past people in several different geo-temporal contexts appear to have converged on bow technology multiple times since the African Middle Stone Age (17, 23, 26–30), the near-synchronous emergence of the bow across vast and ecologically diverse, but contiguous, regions is more parsimoniously interpreted as the product of a successful technological innovation diffusing quickly through cultural transmission networks. If so, this supports cultural evolutionary models that emphasize the ratchet effect of cumulative culture, in which rare innovations are preserved and spread rather than repeatedly reinvented. The single origin versus independent invention hypotheses can be tested as more data become available, but now such testing is beyond current archaeological resolution and analysis.

The contrasting adoption trajectories of bow and atlatl technologies in western North America have broader implications for understanding the wider environmental, ecological, and social contexts of technological evolution. In the south, the nearly instantaneous disappearance of the atlatl following the introduction of the bow exemplifies a process of disruptive innovation. The bow did not simply offer incremental improvements over the atlatl but introduced a novel mechanical principle that, given the context, must have provided substantial functional benefits that rendered the older technology effectively obsolete. This replacement was not gradual but dramatically abrupt, suggesting that once exposed to the new bow technology, the performance improvements over the existing atlatl technology were immediate and obvious to users (even if users are not fully aware of how the technology worked, Ref. 48). Such rapid transitions are consistent with models of punctuated technological change, in which long periods of stability are abruptly interrupted by innovations that open new design spaces and fundamentally alter existing practices. The bow's decisive advantages in accuracy, distance, rate of fire, and versatility, among others, allowed it to displace the atlatl as a one-to-one replacement across diverse southern environments, highlighting how disruptive technologies can quickly homogenize practices over large geographic scales.

In contrast, the prolonged coexistence of the bow and atlatl in the north illustrates a different evolutionary dynamic. Here, rather than replacement, the bow was added to the technological repertoire, resulting in increased toolkit richness. This outcome aligns with risk-buffering hypotheses in which foragers in high-risk, variable environments maintain a wider diversity of tools to ensure flexibility and resilience (15, 49, 50). In northern ecologies, the atlatl may have retained utility during colder months or in the hunting of certain prey, while the bow offered complementary advantages during warmer months or for other prey.

The coexistence of bow and atlatl technologies in the north aligns with a well-documented global trend where hunter-gatherer toolkits generally become more diverse and complex with increasing latitude. This pattern has been demonstrated in multiple comparative studies (49, 50), which show that northern foragers typically maintain a wider range of tools than their tropical counterparts. In environments characterized by high seasonality, unpredictable prey availability, and shorter growing seasons, failure in a single subsistence strategy carries greater consequences for survival, and marked seasonality in resource availability often demands diverse technologies and behaviors. To buffer against this ecological risk, foragers invest in technological diversification rather than relying solely on a single technology. In such contexts, the adoption of a new technology does not necessarily lead to the displacement of older ones. Instead, novel tools are incorporated alongside existing ones, expanding the functional repertoire of the toolkit. This results in what might be described as technological redundancy, where multiple tools address overlapping adaptive challenges but in different ways or with different risk profiles. The atlatl may have remained advantageous for some hunting contexts, while the bow provided complementary strengths in versatility, accuracy, and rapid fire. The coexistence of these technologies therefore reflects a broader strategy of risk mitigation through technological diversification, where redundancy increases resilience in the face of resource variability.

This contrasts sharply with lower-latitude regions, where resource availability is often more stable and predictable. Moreover, while such environments are still seasonal, they are less extreme than at high latitudes. In these high-latitude contexts, the costs of maintaining redundant technologies may outweigh the benefits, favoring optimization where societies converge on a single, most efficient solution to a subsistence challenge. The southern North American pattern, where the bow rapidly and completely replaces the atlatl, seems to illustrate this specialization pathway, whereas the northern pattern, where both persist for centuries, illustrates the diversification pathway.

Taken together, these cases demonstrate that technological evolution is not solely a matter of invention, innovation, and diffusion, but adoption dynamics are shaped by additional factors such as the ecological risk regimes in which societies and their technologies operate. At higher latitudes, technological richness functions as a hedge against uncertainty, while at lower latitudes, technological innovation is more likely to yield rapid replacement. This latitudinal gradient therefore offers a unifying explanatory framework for why some innovations produce additive increases in technological complexity, while others produce disruptive replacement.

## Conclusions

Overall, our analysis shows that bow technology appeared around 1,400 years ago across western North America, but with strikingly different adoption dynamics. In the south, bow technology rapidly displaced the atlatl, while in the north, both technologies coexisted for over a millennium. These results reveal that technological evolution is context dependent, sometimes producing disruptive replacements and at other times enriching toolkits to buffer ecological risk. The atlatl-to-bow transition thus illustrates how innovation, ecology, and cultural transmission combine to shape the evolutionary trajectories of human technological change.

## Materials and methods

### The sample of dated specimens

Our analyses focus on radiocarbon dates assayed from organic weapons in western North America. To be included in the analysis, enough of the dated weapon had to be preserved to allow for a definitive classification of the object as either an atlatl, dart, bow, or arrow (for evaluation of dates, see Supporting Information and Fig. S1). We generated a dataset of 140 radiocarbon dates that directly dated 136 weapons (two artifacts were dated twice and one artifact was dated three times) and were reported in the published literature (data available in Dataset S1). For the three cases where multiple dates were used to date the same specimen, we tested the contemporaneity of the radiocarbon assays using the method of Ward and Wilson (51). In all three cases of multiple dates, the ages were statistically similar, and we used the average of the dates and errors. One atlatl dart foreshaft was dated indirectly, yet securely, via three dates on the organic materials (prairie dog skin and yucca cordage) that made up a bag that contained the dart foreshaft (52). Thus, the total number of dated weapons in the dataset is 136. We excluded 11 dates on 10 additional artifacts that could not be definitively attributed to weapon type because not enough of the weapon was preserved. The dataset includes dates associated with 86 atlatl or dart specimens and dates associated with 50 bows or arrows. There is no reason to think that preservation in glacial ice or in dry caves and rock shelters is biased toward atlatl and dart or bow and arrow. We recorded wood species identifications for weapons when published, with 55 specimens being identified as 16 different species; birch and spruce were the most common (Dataset S1). The north had weapons made from four species, while the south had 13 species of wood represented. Atlatls and darts were made using 10 different species, and bows and arrows from 11 species. Five species were used for both weapon types, including birch, cane, greasewood, willow, and yew.

The sample of dates falls into two spatial clusters, one in the north and one in the south. The northern cluster encompasses parts of the Yukon, Northwest Territories, British Columbia, and Alaska. Grund and Huzurbazar (21) previously analyzed 72 dated weapons (from 74 dates) recovered from glacial ice sheets in the Yukon and Northwest Territories. We expanded their dataset with 14 more dated weapons for a total of 86 dated weapons (from 89 dates) by including recent finds from ice sheets in Alaska and British Columbia, in addition to those previously reported from the Yukon and Northwest Territories (39–42, 53–55). In the northern sample, 53 weapons are classified as atlatl or dart and 33 as bow or arrow. The southern region, in contrast, is a much larger region that extends from Coahuila, Mexico to southern British Columbia, Canada, and from Wyoming to California (Fig. 1). The southern region sample includes 50 dated weapons (from 53 dates), comprising 33 atlatls or darts and 17 bows or arrows. The specimens recovered in the sample from the south are primarily from dry caves and rock shelters and also include a few specimens from ice sheets in British Columbia and Wyoming and a wet site in Washington. The specimens from British Columbia and Washington are the farthest northern extent in the southern region. Their inclusion in the sample does not affect the outcome, as neither specimen dates to the transition period (the arrow from British Columbia is relatively young, dating to 335 cal.BP and the atlatl from Washington is relatively, old dating to 1,700 cal.BP). More dated weapons have been recovered in the north (*n* = 86) compared with the south (*n* = 50); this is due to the abundance of organic materials recovered from melting glacial ice sheets in the Yukon, Northwest Territories, British Columbia, and Alaska. These remains have been recovered through targeted surveys along the melting peripheries of ice sheets (39–42, 53–55).

### Statistical methods

We calibrated the published radiocarbon dates with associated error terms for atlatl, dart, bow, or arrow specimens recovered from western North America using the *rcarbon* package in R 4.4.0 (R Core Team) with the IntCal20 calibration curve (56).

To examine the radiocarbon dates associated with these weapon technologies, we plotted the calibrated radiocarbon date distributions, focusing on the oldest bow and youngest atlatl specimens for each region. In addition, we used an end-to-end Bayesian analysis for summarizing sets of radiocarbon dates in the *baydem* package of R (57). With the end-to-end Bayesian method, we summarize the radiocarbon data separately for both weapon types and regions. This analysis is presented in the Supporting Information.

We used OLE to investigate the timing of the first appearance of bow technology. OLE infers the extinction or first appearance time from a temporal distribution of sightings or, in our case, archaeological finds and has been used recently in archaeology to estimate the timing of different classes of archaeological material (58, 59). OLE is also called the Weibull extreme value model because OLE assumes that the joint distribution of the tail of the distribution (commonly the first 10 or last data points in a distribution) follows a Weibull extreme value distribution to estimate the shape parameter of the distribution using the temporal spread of the observed dates. Hence, the observed times between dated events are crucial.

We ran the OLE function with the *sExtinct* package in R. We bootstrapped 1,000 OLE estimates for the origin of bow technology, separately for the north and south regions. We randomly sampled a single calendar date from each calibrated probability distribution for each bootstrap. We use the median years before present (cal.BP) for each specimen and sample from the probability density functions of each for the OLE analysis. For example, the 33 bow specimens in the north would have 33 sampled calendar dates for each run. Of these, the 10 oldest calendar dates are used to generate an OLE date, and this experiment is repeated 1,000 times, always taking the 10 oldest specimens in the sample. The OLE function generates a distribution of median appearance dates. This bootstrapped distribution is approximately normal, and we use the 95% prediction interval of this distribution to represent the range of calendar dates for the likely first appearance of bow technology in both regions.

While OLE focuses on the timing for the origin of bow technology, we carried out logistic regressions to model the probability of bow versus atlatl use through time. We ran separate models for the north and south because the transition is much steeper (step-like) in the south. We ran Bayesian logistic regression (logit link) implemented in the *brms* package (60, 61) to model the transition from atlatl to bow. The models use calibrated calendar years BP with measurement error (1SD) included in the estimation. We assigned the slope for calendar years BP to have a moderately weak prior probability distribution (*μ* = 0, *σ* = 0.1). Sampling was done using the No-U-Turn Sampler developed by Hoffman and Gelman (62). The final models ran with four chains for 10,000 iterations, each with a warm-up of 5,000 iterations. For all parameters, r-hat values (a model diagnostic with an expected value equal to 1) were exactly 1 and hence signify model convergence. Chains were inspected visually for sufficient mixing to ensure appropriate model results. The model passes a posterior predictive check, and the residuals are well behaved. All code and raw data are available in Dataset S1.

## Supplementary Material

pgag040_Supplementary_Data

## Data Availability

All data are included in Dataset S1.

## References

[1] Harmand S, et al 2015. 3.3-Million-year-old stone tools from Lomekwi 3, West Turkana, Kenya. Nature. 521:310–315. 25993961 10.1038/nature14464

[2] Plummer TW, Harmand S, Finestone EM, Wilson EP. 2025. The first million years of technology: the Lomekwian and the Early Oldowan. Annu Rev Anthropol. 54:359–375.

[3] Arthur WB . The nature of technology: what it is and how it evolves. Simon and Schuster, New York, 2009.

[4] Basalla G . The evolution of technology. Cambridge University Press, Cambridge, UK, 1988.

[5] Whittaker JC . Levers, not springs: how a spearthrower works and why it matters. In: Iovita R, Sano K, editors. Multidisciplinary approaches to the study of stone age weaponry. Springer, Dordrecht, 2016. p. 65–74.

[6] Bebber MR, et al 2024. The gravity of Paleolithic hunting. J Archaeol Sci Rep. 59:104785.

[7] Bergman CA, McEwen E, Miller R. 1988. Experimental archery: projectile velocities and comparison of bow performances. Antiquity. 62:658–670.

[8] Christenson AL . 1986. Projectile point size and projectile aerodynamics: an exploratory study. Plains Anthropol. 31:109–128.

[9] Hughes SS . 1998. Getting to the point: evolutionary change in prehistoric weaponry. J Archaeol Method Theory. 5:345–408.

[10] Lepers C, Rots V. 2020. The important role of bow choice and arrow fletching in projectile experimentation: a ballistic approach. J Archaeol Sci Rep. 34:102613.

[11] Lombard M . 2022. Re-considering the origins of Old World spearthrower-and-dart hunting. Quat Sci Rev. 293:107677.

[12] Raymond A . 1986. Experiments in the function and performance of the weighted atlatl. World Archaeol. 18:153–177.

[13] Tomka SA . 2013. The adoption of the bow and arrow: a model based on experimental performance characteristics. Am Antiq. 78:553–569.

[14] Whittaker JC . 2013. Comparing atlatls and bows: accuracy and learning curve. Ethnoarchaeology. 5:100–111.

[15] Hamilton MJ, Lobo J, Collard M, Walker RS, Buchanan B. 2025. Technological complexity and combinatorial invention in small-scale societies. Sci Adv. 11:eadv6153. 40991691 10.1126/sciadv.adv6153 PMC12459400

[16] Ames KM, Fuld KA, Davis S. 2010. Dart and arrow points on the Columbia Plateau of western North America. Am Antiq. 75:287–325.

[17] Backwell L, et al 2018. The antiquity of bow-and-arrow technology: evidence from Middle Stone Age layers at Sibudu Cave. Antiquity. 92:289–303.

[18] Blitz JH . 1988. Adoption of the bow in prehistoric North America. North Am Archaeol. 9:123–145.

[19] Bradfield J, Lombard M, Reynard J, Wurz S. 2020. Further evidence for bow hunting and its implications more than 60,000 years ago: results of a use-trace analysis of the bone point from Klasies River Main Site, South Africa. Quat Sci Rev. 236:106295.

[20] Castro SC, Marsh E, Yebra L, Cortegoso V. 2024. The origin and dispersion of the bow in the Andes (16–37° S) based on a controlled database of projectile point metrics. Quat Int. 704:82–95.

[21] Grund BS, Huzurbazar SV. 2018. Radiocarbon dating of technological transitions: the Late Holocene shift from atlatl to bow in northwestern Subarctic Canada. Am Antiq. 83:148–162.

[22] Kidder AV . 1938. Arrow-heads or dart points. Am Antiq. 4:156–157.

[23] Langley MC, et al 2020. Bows and arrows and complex symbolic displays 48,000 years ago in the South Asian tropics. Sci Adv. 6:eaba3831. 32582854 10.1126/sciadv.aba3831 PMC7292635

[24] Lombard M, Phillipson L. 2010. Indications of bow and stone-tipped arrow use 64,000 years ago in KwaZulu-Natal, South Africa. Antiquity. 84:635–648.

[25] Maschner H, Mason OK. 2013. The bow and arrow in northern North America. Evol Anthropol. 22:133–138. 23776050 10.1002/evan.21357

[26] Metz L, Lewis JE, Slimak L. 2023. Bow-and-arrow, technology of the first modern humans in Europe 54,000 years ago at Mandrin, France. Sci Adv. 9:eadd4675. 36812314 10.1126/sciadv.add4675 PMC9946345

[27] Rosendahl G, et al 2006. Le plus vieil arc du monde? Une pièce intéressante en provenance de Mannheim, Allemagne. L’Anthropologie. 110:371–382.

[28] Sano K . 2016. Evidence for the use of the bow-and-arrow technology by the first modern humans in the Japanese islands. J Archaeol Sci Rep. 10:130–141.

[29] Thomas DH . 1978. Arrowheads and atlatl darts: how the stones got the shaft. Am Antiq. 43:461–472.

[30] Weinstock J . 2000. Demography through osteometry: sex ratios of reindeer and hunting strategies in the Late Glacial site of Stellmoor, northern Germany. Archaeozoologia. 11:187–198.

[31] Bettinger RL . 2013. Effects of the bow on social organization in Western North America. Evol Anthropol. 22:118–123. 23776048 10.1002/evan.21348

[32] Bettinger RL, Eerkens J. 1999. Point typologies, cultural transmission, and the spread of bow-and-arrow technology in the prehistoric Great Basin. Am Antiq. 64:231–242.

[33] Blitz JH, Porth ES. 2013. Social complexity and the bow in the Eastern Woodlands. Evol Anthropol. 22:89–95. 23776044 10.1002/evan.21349

[34] Lyman R, VanPool TL, O’Brien MJ. 2009. The diversity of North American projectile-point types, before and after the bow and arrow. J Anthropol Archaeol. 28:1–13.

[35] Milner GR, Chaplin G, Zavodny E. 2013. Conflict and societal change in late prehistoric eastern North America. Evol Anthropol. 22:96–102. 23776045 10.1002/evan.21351

[36] Reed PF, Geib PR. 2013. Sedentism, social change, warfare, and the bow in the ancient Pueblo Southwest. Evol Anthropol. 22:103–110. 23776046 10.1002/evan.21356

[37] Buchanan B, Hamilton MJ, Walker RS. 2025. A new method for classifying dart and arrow projectile points. Am Antiq. 91:168–182. 10.1017/aaq.2025.10109

[38] Hildebrandt WR, King JH. 2012. Distinguishing between darts and arrows in the archaeological record: implications for technological change in the American West. Am Antiq. 77:789–799.

[39] Alix C, Hare PG, Andrews TD, MacKay G. 2012. A thousand years of lost hunting arrows: wood analysis of ice patch remains in northwestern Canada. Arctic. 65:95–117.

[40] Andrews TD, MacKay G, Andrew L. 2012. Archaeological investigations of alpine ice patches in the Selwyn Mountains, Northwest Territories, Canada. Arctic. 65:1–21.

[41] Hare PG, Thomas CD, Topper TN, Gotthardt RM. 2012. The archaeology of Yukon ice patches: new artifacts, observations, and insights. Arctic. 65:118–135.

[42] Thomas CD, et al 2023. The Alligator Lake throwing dart, Yukon, Canada. J Glacial Archaeol. 7:25–45.

[43] Whittaker JC . 2012. Ambiguous endurance: late atlatls in the American Southwest? Kiva. 78:79–98.

[44] Amick DS . Technological organization and the structure of inference in lithic analysis: an examination of Folsom hunting behavior in the American Southwest. In: Carr PJ, editor. The organization of North American prehistoric chipped stone tool technologies. International Monographs in Prehistory, Ann Arbor, MI, 1994. p. 9–34.

[45] Geib PR . 1989. Implications of early bow use in Glen Canyon. Utah Archaeol. 2:32–47.

[46] Roth BJ, Toney E, Lorentzen L. 2011. The advent of bow and arrow technology in the Mimbres Mogollon region. Kiva. 77:87–109.

[47] Yohe RM . 1998. The introduction of the bow and arrow and lithic resource use at Rose Spring (CA-INY-372). J Calif Great Basin Anthropol. 20:26–52.

[48] Harris JA, Boyd R, Wood BM. 2021. The role of causal knowledge in the evolution of traditional technology. Curr Biol. 31:1798–1803.e3. 33631097 10.1016/j.cub.2021.01.096

[49] Collard M, Buchanan B, Morin J, Costopoulos A. 2011. What drives the evolution of hunter–gatherer subsistence technology? A reanalysis of the risk hypothesis with data from the Pacific Northwest. Philos Trans R Soc Lond B Biol Sci. 366:1129–1138. 21357235 10.1098/rstb.2010.0366 PMC3049102

[50] Collard M, Buchanan B, O’Brien MJ, Scholnick J. 2013. Risk, mobility or population size? Drivers of technological richness among contact-period western North American hunter–gatherers. Philos Trans R Soc Lond B Biol Sci. 368:20120412. 24101622 10.1098/rstb.2012.0412 PMC4027409

[51] Ward GK, Wilson SR. 1978. Procedures for comparing and combining radiocarbon age determinations: a critique. Archaeometry. 20:19–31.

[52] Geib PR . 2004. AMS dating of a Basketmaker II hunter's bag (cache 1) from Sand Dune Cave, Utah. Kiva. 69:271–282.

[53] Dixon EJ, Manley WF, Lee CM. 2005. The emerging archaeology of glaciers and ice patches: examples from Alaska's Wrangell–St. Elias National Park and Preserve. Am Antiq. 70:129–143.

[54] VanderHoek R, Tedor RM, McMahan JD. 2007. Cultural materials recovered from ice patches in the Denali Highway region, central Alaska, 2003–2005. Alaska J Anthropol. 5:185–200.

[55] VanderHoek R, Wygal B, Tedor RM, Holmes CE. 2007. Survey and monitoring of ice patches in the Denali Highway region, central Alaska, 2003–2005. Alaska J Anthropol. 5:67–86.

[56] Reimer PJ, et al 2020. The IntCal20 Northern Hemisphere radiocarbon age calibration curve (0–55 cal kBP). Radiocarbon. 62:725–757.

[57] Price MH, et al 2021. End-to-end Bayesian analysis for summarizing sets of radiocarbon dates. J Archaeol Sci. 135:105473.

[58] Key AJ, Roberts DL, Jarić I. 2021. Statistical inference of earlier origins for the first flaked stone technologies. J Hum Evol. 154:102976. 33773284 10.1016/j.jhevol.2021.102976

[59] Key A, et al 2024. Identifying accurate artefact morphological ranges using optimal linear estimation: method validation, case studies, and code. J Archaeol Sci. 162:105921.

[60] Bürkner P-C . 2017. brms: an R package for Bayesian multilevel models using Stan. J Stat Softw. 80:1–28.

[61] Bürkner P-C . 2018. Advanced Bayesian multilevel modeling with the R package brms. R J. 10:395–411.

[62] Hoffman MD, Gelman A. 2014. The no-U-turn sampler: adaptively setting path lengths in Hamiltonian Monte Carlo. J Mach Learn Res. 15:1593–1623.

