# Supplementary material for: Rapid adoption of bow technology across western North America ∼1,400 years ago
Source: PNAS Nexus. 2026 Mar 17;5(3):pgag040. doi: 10.1093/pnasnexus/pgag040 (PMC12993813; doi:10.1093/pnasnexus/pgag040)
Supplement: pgag040_Supplementary_Data [file pgag040_supplementary_data.zip › PNASNEXUS-PNASNEXUS-2025-01721-TRR-s01.pdf]

Supporting Information for

Rapid adoption of bow technology across western North America ~1,400 years ago

Briggs Buchanan[1], Marcus J. Hamilton[2,3], Metin I. Eren[4,5,6], and Robert S. Walker[7]

1. Department of Anthropology & Sociology, University of Tulsa, Tulsa, Oklahoma, U.S.A.
2. Department of Anthropology & School of Data Science, University of Texas at San Antonio, San Antonio, Texas, U.S.A.
3. Santa Fe Institute, Santa Fe, New Mexico, U.S.A.
4. Department of Anthropology, Kent State University, Kent, Ohio, U.S.A.
5. Cleveland Museum of Natural History, Cleveland, Ohio, U.S.A.
6. McDonald Institute for Archaeological Research, University of Cambridge, Cambridge, U.K.
7. Department of Anthropology, University of Missouri, Columbia, Missouri, U.S.A.

* corresponding author: Briggs Buchanan.

Email: briggs-buchanan@utulsa.edu

This PDF file includes:

Supporting text
Figure S1
Figure S2
SI References

Other supporting materials for this manuscript include the following:

Dataset S1 (separate file). Buchanan et al. – Atlatl and Bow C14 dataset.

R Code. Buchanan et al. Dating atlatls and bows.

Supporting Information Text

Evaluation of radiocarbon date data

We vetted the radiocarbon dates prior to further analyses of our date compilation. We emphasize that because this study relies on dates derived from organic wood weapons (except for the one weapon dated indirectly via the skin bag the weapon was contained within) the primary issue of association between date and the intended object or event to be dated is not an issue for our study. Otherwise, we follow Collard et al.'s (S1) conservative approach to date selection. We used only dates that were deemed valid by the originating laboratory and researchers. We also included dates with large standard errors. This approach is conservative because the inclusion of imprecise dates can be expected to diminish any differences between atlatl-dart and bow-arrow age ranges. Examining the standard deviations associated with the 136 dates in our dataset shows they range from 23-350, but the distribution is skewed (skewness 4.43) with a median error of 40 years (Figure S1). Moreover, large standard deviations or errors are usually associated with legacy dates and only four radiocarbon dates in our dataset have errors greater than 150 years (these errors are 180, 185, 200, 350).

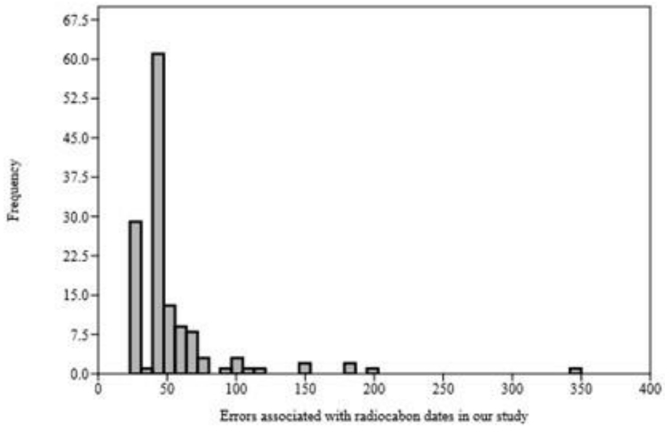

Figure S1. Histogram of one-sigma errors associated with radiocarbon dates on atlatl and darts and bow and arrows in the Buchanan et al. study.

We evaluated the published isotopic values associated with the dates in our dataset (see supplemental data table). Isotopic values are used by radiocarbon labs to normalize results and correct for isotopic fractionation. In addition, isotopic values can be useful in checking the validity of radiocarbon dates, but unfortunately it is not common for values to be published along with radiocarbon ages. We searched for isotopic values associated with the 140 dates in our study and found 36 had published isotope values and added these values to the supplementary data table. According to Philippsen et al. (S2) the typical and "good" range for wood from most terrestrial plants (C3 plants) is approximately -20‰ to -30‰, with an average value of -25‰ relative to the Vienna Pee Dee Belemnite (VPDB) standard. The isotope values on the wood specimens in our dataset average -23.53‰, thus within the acceptable range for wood specimens. Another four isotopic values in our data were from materials other than wood, including agave stalk (-8.1), and prairie dog skin and yucca (-22.5, -21.6, -9.1).

End-to-end Bayesian analysis summarizing the atlatl and dart and bow and arrow radiocarbon data for the north and south regions

Statistical Methods. To analyze the radiocarbon dates associated with these weapon technologies, we used an end-to-end Bayesian analysis for summarizing sets of radiocarbon dates in the *baydem* package of R (S3). This method avoids many of the well-known biases and summary problems associated with summed probability distribution methods (S3-S6). With the end-to-end Bayesian method, we summarize the radiocarbon data separately for both weapon types and regions. We modeled the number of components in the finite Gaussian mixture models iteratively from two to seven. The *baydem* package has a built-in approach to evaluate the best model to obtain the optimal number of mixture components based on the Pareto smoothed importance sampling approximation of the leave-one-out cross-validation (S7).

Results. Summarizing the radiocarbon dates for atlatl and dart and bow and arrow by region using the end-to-end Bayesian analysis shows the overlap between atlatl and bow technology in the north and the rapid transition in the south (Figure S2). Also shown in Figure S2 is the OLE estimates and the associated 95% uncertainty associated with these estimates. The estimate for the origin of the bow and arrow is more uncertain for the south. However, for both regions the average OLE estimates closely align with the timing of the transition identified in the summarized radiocarbon dates.

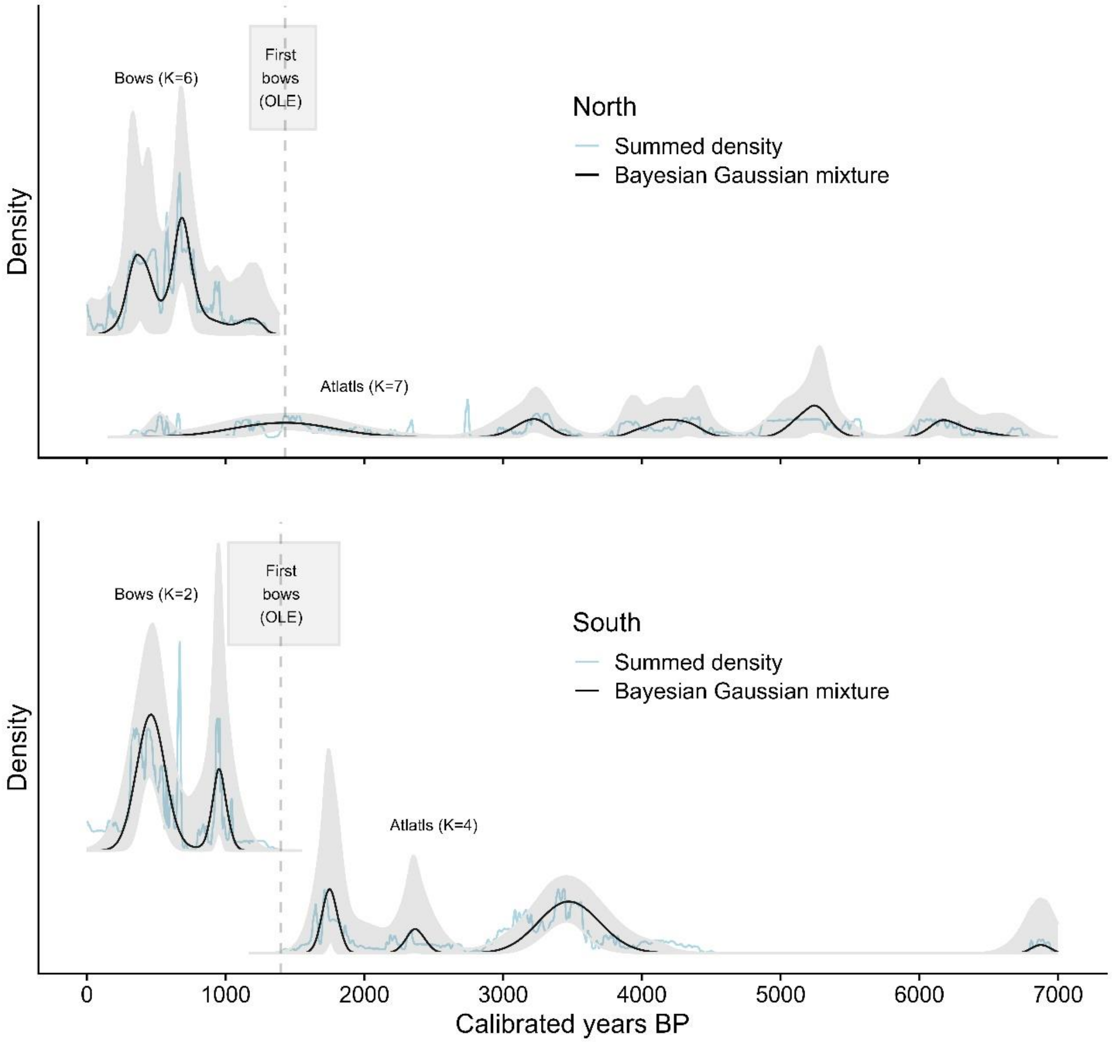

Figure S2. Results of the end-to-end Bayesian analysis summarizing the radiocarbon assays associated with (A) the north and (B) the south regions of western North America. The plots show the summarized radiocarbon data for the summed density (blue) and the Bayesian Gaussian mixture model (black) with uncertainty bands (gray). The number of components (K) for the finite Gaussian mixture models used for each weapon type and region was determined iteratively. The 95% confidence interval surrounding the Optimal Linear Estimates (OLE) for the origin of bow and arrow in each region is shown with gray boxes.

Dataset S1 (separate file). Buchanan et al. – Atlatl and Bow C14 dataset.

R Code. Buchanan et al. Dating atlatls and bows.

SI References

S1. M. Collard, K. Edinborough, S. Shennan, M. G. Thomas, Radiocarbon evidence indicates that migrants introduced farming to Britain. *J. of Archaeol. Sci.* **37**, 866-870 (2010).

S2. B. Philippsen, J. Olsen, S. A. Sørensen, B. Måge, δ13C values of wood and charcoal reveal broad isotopic ranges at the base of the food web. *Radiocarbon* **61**, 2003-2017 (2019).

S3. M. H. Price, J. M. Capriles, J. A. Hoggarth, R. K. Bocinsky, C. E. Ebert, J. H. Jones, End-to-end Bayesian analysis for summarizing sets of radiocarbon dates. *J. Archaeol. Sci.* **135**, 105473 (2021).

S4. V. Attenbrow, P. Hiscock, Dates and demography: Are radiometric dates a robust proxy for long-term prehistoric demographic change? *Archaeol. Oceania* **50**, 30–36 (2015).

S5. L. Becerra-Valdivia, R. Leal-Cervantes, R. Wood, T. Higham, Challenges in sample processing within radiocarbon dating and their impact in ^{14}C-dates-as-data studies. *J. Archaeol. Sci.* **113**, 105043 (2020).

S6. W. A. Brown, Through a filter, darkly: Population size estimation, systematic error, and random error in radiocarbon-supported demographic temporal frequency analysis. *J. Archaeol. Sci.* **53**, 133–147 (2015).

S7. D. A. Contreras, J. Meadows, Summed radiocarbon calibrations as a population proxy: A critical evaluation using a realistic simulation approach. *J. Archaeol. Sci.* **52**, 591–608 (2014).

S8. A. Vehtari, A. Gelman, J. Gabry, Practical Bayesian model evaluation using leave-one-out cross-validation and WAIC. *Stat. Comput.* **27**, 1413–1432 (2017).
